# Loss of Opi3 causes a lipid imbalance that influences the virulence traits of *Cryptococcus neoformans* but not cryptococcosis

**DOI:** 10.3389/fcimb.2024.1448229

**Published:** 2024-08-13

**Authors:** Christopher W. J. Lee, Anna Brisland, Xianya Qu, Linda C. Horianopoulos, Guanggan Hu, François L. Mayer, James W. Kronstad

**Affiliations:** ^1^ Michael Smith Laboratories, University of British Columbia, Vancouver, BC, Canada; ^2^ Department of Microbiology and Immunology, University of British Columbia, Vancouver, BC, Canada

**Keywords:** fungal pathogenesis, choline, phospholipids, cell wall, Kennedy pathway

## Abstract

The basidiomycete fungus *Cryptococcus neoformans* is a useful model for investigating mechanisms of fungal pathogenesis in mammalian hosts. This pathogen is the causative agent of cryptococcal meningitis in immunocompromised patients and is in the critical priority group of the World Health Organization fungal priority pathogens list. In this study, we employed a mutant lacking the *OPI3* gene encoding a methylene-fatty-acyl-phospholipid synthase to characterize the role of phosphatidylcholine (PC) and lipid homeostasis in the virulence of *C. neoformans*. We first confirmed that *OPI3* was required for growth in nutrient limiting conditions, a phenotype that could be rescued with exogenous choline and PC. Additionally, we established that loss of Opi3 and the lack of PC lead to an accumulation of neutral lipids in lipid droplets and alterations in major lipid classes. The growth defect of the *opi3Δ* mutant was also rescued by sorbitol and polyethylene glycol (PEG), a result consistent with protection of ER function from the stress caused by lipid imbalance. We then examined the impact of Opi3 on virulence and found that the dependence of PC synthesis on Opi3 caused reduced capsule size and this was accompanied by an increase in shed capsule polysaccharide and changes in cell wall composition. Further tests of virulence demonstrated that survival in alveolar macrophages and the ability to cause disease in mice were not impacted by loss of Opi3 despite the choline auxotrophy of the mutant *in vitro*. Overall, this work establishes the contribution of lipid balance to virulence factor elaboration by *C. neoformans* and suggests that host choline is sufficient to support proliferation during disease.

## 1 Introduction

Fungal pathogens cause severe diseases in animals and plants, and therefore have substantial impacts on human health and agriculture (Fisher et al., 2020). The fungal pathogen *Cryptococcus neoformans* is well known for causing cryptococcal meningitis in immunocompromised individuals and is on the fungal priority pathogens list of the World Health Organization alongside *Candida albicans*, *Aspergillus fumigatus*, and *Candida auris* (WHO, 2022; Fisher and Denning, 2023). In fact, *C. neoformans* is estimated to cause 152,000 annual cases of meningoencephalitis globally each year with 112,000 cases resulting in mortality (Rajasingham et al., 2022). Several factors contribute to the ability of this pathogen to cause disease in humans including thermotolerance at 37°C, the production of melanin in the cell wall, and the formation of an elaborate polysaccharide capsule (Casadevall et al., 2000; Zaragoza et al., 2009; Alspaugh, 2015). The molecular mechanisms that underpin these virulence attributes are not completely understood and the subject of active investigation.

Several studies are beginning to assess the role of phospholipid metabolism in the virulence of *C. neoformans* including identification of the major phospholipid subclasses: phosphatidylcholine (45%, PC), phosphatidylethanolamine (20%, PE), and phosphatidylserine (12%, PS) (Singh et al., 2017). The remaining pool of phospholipids is comprised of phosphatidylinositol (PI), phosphatidylglycerol (PG), cardiolipin (CL), and phosphatidic acid (PA) (Shea et al., 2006; Longo et al., 2015; Singh et al., 2017). As in the model yeast *Saccharomyces cerevisiae* and the human pathogenic yeast *Candida albicans*, the synthesis of phospholipids can occur via a *de novo* cytidinediphosphate diacylglycerol (CDP-DAG) pathway or via the Kennedy pathway using exogenous choline or ethanolamine (Konarzewska et al., 2019; Tams et al., 2019). It is known in *S. cerevisiae* that phospholipids are asymmetrically distributed in the plasma membrane bilayer such that PC is known to predominate in the exoplasmic leaflet and PE and PS are found on the cytosolic leaflet (Mioka et al., 2014). Several studies have examined the roles of the flippases in membrane asymmetry in *C. neoformans* (Hu et al., 2017; Rizzo et al., 2018). For example, work on Apt1, a P-type ATPase belonging to the type IV, Drs2 family of flippases (aminophospholipid translocases), and Cdc50, a noncatalytic subunit of type IV P‐type ATPases, in C. *neoformans* has shown that lipid distribution in the membrane impacts echinocandin sensitivity, membrane and cell wall integrity, and survival in a mouse model of cryptococcosis (Huang et al., 2016; Hu et al., 2017).

Connections between phospholipid homeostasis and fungal virulence are emerging. For example, overexpression of the Kennedy pathway in *C. albicans* leads to an accumulation of PE/PC causing hypervirulence in a mouse model of candidiasis hinting to a greater role for these two phospholipids in virulence (Tams et al., 2019). Moreover, phosphatidylserine is an essential phospholipid for cryptococcal viability, and phospholipids are known to influence capsule size when *C. neoformans* cells are interacting with amoebae and macrophages (Chrisman et al., 2011; Konarzewska et al., 2019). These studies suggest that additional studies on the role of PC, as the most abundant glycerolipid in the fungal lipid bilayer, may provide novel insights into the role of lipid homeostasis in the virulence of *C. neoformans*.

In fungi, PC can be synthesized via the CDP-diacylglycerol *de novo* pathway, or via the Kennedy pathway which makes use of exogenous choline (Gibellini and Smith, 2010). As mentioned, PS, as a precursor of PC is a known essential component for *Cryptococcus* viability (Konarzewska et al., 2019). To investigate the role of choline and PC in *C. neoformans* virulence, we extended our previous study which identified the *OPI3* gene in a screen for mutants sensitive to lithium chloride (Mayer et al., 2018). *OPI3* encodes a methylene-fatty-acyl-phospholipid synthase responsible for the final methylation step in the biosynthesis of PC from PE. Previous work on Opi3 in *S. cerevisiae* showed that cells lacking Opi3 are deficient in PC and cannot enter log phase due to phospholipid defects in the plasma membrane (McGraw and Henry, 1989). More recently, inhibition of PC synthesis in yeast was shown to cause changes in phospholipid levels, impaired growth, changes in ER and mitochondrial structure, the accumulation of lipid droplets, and activation of the unfolded protein response (Vevea et al., 2015). In our current study, we characterized an *opi3Δ* mutant of *C. neoformans* to study the role of PC in cryptococcal virulence and capsule production. An *opi3Δ* mutant could not grow *in vitro* without supplemental choline or PC and exhibited growth defects on minimal media. Interestingly, growth could be rescued with sorbitol and polyethylene glycol thus identifying a choline-independent mechanism to maintain cell viability that may involve support for ER function. Moreover, the *opi3Δ* mutant accumulated lipid droplets and had reduced levels of PE, PS, PA, PG, and PC compared to the wild-type strain. We show that the *OPI3* gene is important for capsule formation and cell wall stability although, remarkably, no effect on virulence was observed in a mouse model of cryptococcosis. We attribute the lack of a virulence defect for the mutant in mice to the ability of *C. neoformans* to use host sources of choline to replenish the PC in the plasma membrane.

## 2 Materials and methods

### 2.1 Strains and growth conditions

*C. neoformans* var. *grubii* strain H99 (serotype A) was used as the wild-type (WT) strain. Fungal strains were routinely maintained on YPD agar (1% yeast extract, 2% bacto-peptone, 2% D-glucose, 2% agar). Overnight cultures were grown in 5 ml of liquid YPD medium in a shaking incubator at 30°C and 180 rpm. All modified strains were constructed by biolistic transformation of linear constructs generated by the amplification of a construct from an existing mutant background or via a split-marker design. The primers used for strain construction are listed in **Supplementary Table S1**. The *opi3Δ* deletion strain was constructed in the H99 strain background using the *opi3Δ* construct from the collection of deletion mutants (Chun and Madhani, 2010) (KN99α background). YPD agar containing nourseothricin (NAT; 100 μg/ml) was used to select transformants deleted for the *OPI3* gene. Two independent mutants were confirmed by PCR of the upstream and downstream flanking regions. The strain expressing an Opi3 fusion protein with GFP (C-terminally tagged) was generated in the H99 background using a neomycin resistance cassette and the GFP gene from the plasmid pWH091, as described previously (Jung et al., 2008). Solid YPD medium containing 200 μg/ml G418 (NEO) was used to select the transformants expressing Opi3‐GFP. The construct was introduced into the native locus of the *opi3Δ* deletion strain to complement the mutation resulting in the strain designated *opi3Δ*::*OPI3-GFP*. Anti-GFP antibody (Sigma Aldrich) was used to detect Opi3-GFP by immunoblotting (**Supplementary Figure S1**).

### 2.2 Opi3 localization

Localization of Opi3-GFP was evaluated along with 4’,6-diamidino-2-phenylindole (DAPI) staining from cultures grown overnight in YNB + 2% glucose media. Cells were stained in 10 μg/ml DAPI for 30 minutes in the dark and washed 3 times with phosphate buffered saline (PBS) (Gibco, Waltham, MA, United States) before imaging with a Leica STELLARIS 5 confocal microscope equipped with laser lines for 405, 488 nm and a HC PL APO 63x/1.40 OIL CS2 objective. The LAS X Lightning Expert software was used for additional image deconvolution. Images were processed in ImageJ.

### 2.3 Growth analysis

The influence of choline and phospholipids on growth was assayed with YNB minimal medium (1x YNB, 2% glucose, pH 5.6) supplemented with choline (10 μM), ethanolamine (500 μM), or PC (300 μM). Growth assays using serum were also performed with YNB minimal media but supplemented with either 5% serum extracted from BALB/C mice or 5% heat-inactivated fetal bovine serum (Fetal Bovine Serum, certified, Gibco™, United States). Starting cell densities of 100,000 cells/ml were determined by hemocytometer (Neubauer) counting. Growth assays with cells in 96-well plates were maintained at 30°C with shaking at 180 rpm and OD_600_ readings were taken in 12 or 24 h increments. A microplate reader (Infinite M200, Tecan) was used for automated growth curve analyses. Sensitivity to cell wall and osmotic stressors was assessed on solid media using 10-fold serial dilutions of cells spotted onto YNB agar supplemented with 1 M sorbitol, 1 M NaCl, 1 mg/ml calcofluor white, or 0.5 mg/ml congo red. For spot assays, cells were grown overnight in YPD, washed twice in distilled water, diluted to 20,000 cells/μl, 10-fold serially diluted, and spotted on plates in 5 μl volumes.

### 2.4 Analysis of melanin formation

Secreted melanin was assessed via a liquid growth assay with media containing 0.1% L-asparagine, 0.1% dextrose, 3 mg/ml KH_2_PO_4_, 0.25 mg/ml MgSO_4_∙7H_2_O, 1 μg/ml thiamine, 5 ng/ml biotin, and 0.2 mg/ml L-3,4-dihydroxyphenylalanine (L-DOPA). After overnight growth in YPD, 5 x 10^5^ cells were transferred to 5 ml of L-DOPA media (described above) with and without additional treatments (choline, sorbitol, polyethylene glycol) for 72 h at 30°C. Subsequently, 1 ml of cells was centrifuged before transferring 100 μl of supernatant to a 96 well plate for secreted melanin quantification at OD_490_ via a plate reader. The remaining cell pellet was washed 2x with sterile distilled water before being digested with 300 μl of 1 N NaOH with 10% DMSO at 95C for 1 h to measure extracted melanin content. After centrifuging the digestion mixture, 100 μl of the supernatant was measured at OD_490_ similarly to the secreted melanin. All measurements were normalized to the cell counts of each culture to account for differences in cell number.

### 2.5 Capsule formation

To investigate capsule formation, stationary phase cultures from overnight incubation in YPD were washed twice in low-iron media (LIM, described below) and counted on a hemocytometer. New cultures were inoculated at 5x10^6^ cells/ml in low-iron capsule induction medium (LIM; 5 g L-1 glucose, 5 g L-1 L-asparagine, 0.4 g L-1 K_2_HPO_4_, 0.25 g L-1 CaCl_2_ x 2 H_2_O, 0.08 g L-1 MgSO_4_ x 7H_2_O, 4.78 g L-1 HEPES, 1.85 g L-1 NaHCO_3_; dissolved in chelex 100 resin-treated water, pH 7.4) supplemented with or without choline chloride or PC. Samples were incubated at 30°C and 180 rpm for 48 h. Fungal cells were then stained with India ink at a 1:1 ratio to visualize the polysaccharide capsule by DIC microscopy. Cells were imaged using a Zeiss Plan-Apochromat 100x/1.46 oil lens on a Zeiss Axioplan 2 microscope. Images were obtained using an ORCA-Flash4.0 LT digital CMOS camera (Hamamatsu, Hamamatsu City, Japan).

### 2.6 Capsule shedding assay

The amount of shed capsule polysaccharide in the medium was assessed after 48 h of growth in LIM as previously described (77). Briefly, cells were grown in YPD overnight and washed three times with low-iron water. Cells were transferred to 5 ml of LIM at a starting density of 2.5 x 10^6^ cells/ml and incubated at 30°C for 48 h with or without 10 μM choline chloride, 300 μM PC, or 1 M sorbitol. Supernatant from each sample was normalized to an OD_600_ = 1 to control for changes in cell density, denatured at 70°C for 15 min, subjected to electrophoresis on an agarose gel (60 volt for 3h at room temperature), and blotted onto a nylon membrane (GE Healthcare, Mississauga, ON, Canada) overnight. The membrane was incubated with a 1:5,000 dilution of the 18b7 monoclonal antibody, followed by incubation with a 1:5,000 dilution of anti-mouse HRP (Bio-Rad). Bound polysaccharide was visualized by chemiluminescence (GE Healthcare, Mississauga, ON, Canada). Quantification of the band intensity was performed by taking the peak area of each band using ImageJ software and normalizing each blot to the background intensity.

### 2.7 Analysis of lipid droplets

To investigate changes in total lipid (TAG) content between strains, cells were grown overnight in YNB and supplemented with or without 10 μM choline or 1.5 M sorbitol for 24 or 48 h. Before staining, cells were standardized to a minimum OD_600_ = 1. Cells were washed twice in PBS and stained with the lipophilic dye Nile Red (552/636 nm) (Invitrogen) at a working concentration of 2 µg/ml for 30 min in the dark at room temperature. Stained cells were washed 3 times in PBS prior to mounting 8 μl on a glass slide for fluorescent imaging. Images were taken on a Zeiss Axioplan 2 microscope using a Zeiss Plan-Apochromat 100x/1.46 oil lens. The Nile red channel was used at an exposure of 150 ms for all samples. A second set of samples were prepared and kept on ice for flow cytometry analysis. Lipid droplet size was measured using ImageJ software by taking the average diameter of at least 30 cells per sample. Flow cytometric measurements were performed using CytoFLEX S (Beckman Coulter) Flow Cytometer equipped with four laser lines (405 nm, 488 nm, 561 nm and 633 nm) fitted with a PE (585/42) filter. The number of cells measured per experiment was set to 30,000 unless otherwise stated. Three independent experiments were completed to support statistical analysis. Results were analyzed using FlowJo10.8.1 and gating was made to highlight a single-cell population. The distribution of each sample against the PE filter were plotted compared to highlight differences in total lipid content.

### 2.8 Lipidomic quantification

Cells were grown in YNB with or without 10 μM choline chloride and 1 x 10^7^ cells were frozen on dry ice. Cells were processed at the University of Victoria Genome BC Proteomics Centre for quantification of PC, PE, PS, diacylglyceryl-N,N,N-trimethylhomoserine, and diacylgyceryl hydroxymethyl-N,N,N-trimethyl-beta-alanine. Each cell pellet sample was suspended in 200 μL of methanol and 100 μL of water and two 3-mm metal beads were added for lipid extraction. The samples were lysed on a MM 400 mill mixer at 30 Hz for 2 min. 400 μL of methanol and 300 μL of chloroform were then added. The samples were sonicated in a water bath for 2 min, followed by centrifugation at 21,000 g for 10 min. The clear supernatants were collected to a set of 1.5-mL Eppendorf tubes and the protein pellets were used for protein assay using a standardized Bradford procedure according to the manufacturer’s instructions.

Serially diluted solutions of lipid standards (PC(18:1/18:1), PE(18:0/18:1(9Z)), PG(16:0/18:1(9Z)), PA(18:0/18:0) and PS(16:0/16:0)) were prepared in an internal standard solution of PC(17:0/18:1)-d5, PA(18:0/18:0)-d5, PE(17:0/18:1)-d5, PS(17:0/18:1)-d5 and PG(17:0/18:1)-d5, dissolved in methanol-chloroform (1:1). 200 μL of the supernatant of each sample was dried under a nitrogen gas flow. The residue was dissolved in 200 μL of the same internal standard solution. 5μL aliquots of each sample solution and each standard solution were injected into a C8 LC column (2.1*50 mm, 2.5μm) to make two rounds of UPLC-MRM/MS on a Thermo Ultimate 3000 UHPLC system connected to a Sciex QTRAP 6500 Plus mass spectrometer operated in the positive-ion mode for detection and quantitation of PC and PE lipids and in the negative-ion mode for detection of PA, PG and PS lipids. Chromatographic separations were performed with a mobile phase composing of 5-mM ammonium acetate in water (solvent A) and acetonitrile-isopropanol (1:1) (solvent B) for binary solvent gradient elution. The efficient gradient was 30% to 100% B over 15 min, at 0.4 mL/min and 55°C, followed by 100% B for 3 min and 3-min column equilibrium between injections. Linear-regression calibration curves of the individual lipids were constructed with the data acquired from injections of the lipid standard solutions. Concentrations of detected individual lipids of each class were calculated by interpolating the calibration curve of the lipid belonging to the same class [e.g., the concentrations of the detected PC lipids were calculated using the calibration curves of PC (18:1/18:1)] with the peak area ratio measured from injections of the sample solutions. Concentrations were normalized to total protein concentration and statistics were completed in R (version 4.1.3).

### 2.9 Filipin staining

Cells were grown in YPD overnight and transferred to YNB for 24 h with or without choline at 30°C. Cell number was standardized between samples to an OD_600_ = 1 washed 3 times with PBS before adding filipin (Gibco, Waltham, MA, United States) at a final concentration of 2 µg/ml for 5 min in the dark at room temperature. The stained cells were washed 3x with PBS before mounting on a glass slide. Imaging employed a Zeiss Axioplan 2 fluorescent microscope using a Zeiss Plan-Apochromat 100x/1.46 oil lens. The DAPI channel was used at an exposure of 150ms for all samples. All fluorescent images were processed using Zen 3.0 software (Zeiss, Oberkochen, Germany) and ImageJ to generate intensity profiles across the cell diameter.

### 2.10 RNA extraction and RT-qPCR

Cultures were grown overnight in 5 ml of YPD cultures and transferred to YNB minimal media with and without 1 M sorbitol or 10% polyethylene glycol for 24 h prior to total RNA extractions using a RNeasy kit (Qiagen) following the manufacturer’s instructions for yeast with mechanical disruption. Total RNA was treated with Turbo DNase (Ambion, Austin, TX, United States) according to the manufacturer’s instructions and cDNA was synthesized using the Verso cDNA reverse transcription kit using oligo(dT) to select for polyadenylated mRNA (Thermo Fisher, Waltham, MA, USA). RT-qPCR was performed using Green-2-Go qPCR Mastermix Low ROX (Bio Basic, Amherst, NY, United States) with the primers listed in **Supplementary Table S2**. The reactions were performed on an Applied Biosystems 7,500 Fast real-time PCR system.

### 2.11 Quantification of cell wall components

Strains were grown for 24 h in YNB at 30°C before normalizing cell densities to an OD_600_ = 1. Chitin was stained with 100 μg/ml calcofluor white (CFW) in PBS while chitosan was stained with 250 μg/ml eosin Y in McIlvaine’s buffer pH 6.0 for 15 min in the dark as previously described (Santiago-Tirado et al., 2015; Horianopoulos et al., 2021). Surface exposed β-1,3-glucans were measured using 0.05% aniline blue staining for 10 minutes, as previously described (O’Meara et al., 2013). Samples were washed 3x with PBS post staining and analyzed on an Attune Nxt Flow Cytometer (Invitrogen, Carlsbad, CA) using a FITC filter for Eosin Y and a PB450 filter for CFW and aniline blue. Data were analyzed using FlowJo v10.8.1 software (FlowJo, LLC, Ashland, OR, United States) while statistical significance was determined by performing an ANOVA with Tukey’s multiple comparisons in R (version 4.1.3).

### 2.12 Transmission electron microscopy

Cells were grown in YNB for 24 h at 30°C before normalizing to OD_600_ nm=1. Cells were washed 3x in PBS before fixing with 2.5% glutaraldehyde in 0.1M sodium cacodylate buffer (pH = 6.9) for 1 h at room temperature. Fixative solution was removed by washing with 0.1 M sodium cacodylate buffer three times. After fixation, cells were separated in 3% low temperature gelling agarose and post fixed 1% OsO4, 0.8% K_4_[Fe(CN)_6_], 5 mM CaCl_2_ in 0.1 M sodium cacodylate at pH7.4 for 10 min. The cells were washed three times with ddH_2_O and incubated in 1% thiocarbohydrazide (TCH) in dH_2_O for 5 min and then washed three times in dH_2_O and then post-fixed again for 2 min then washed 3x in dH2O. Samples were dehydrated through sequential washes with a graded concentration series of acetone into 100% ethanol. After dehydration, cells were embedded in Spurr’s resin and 70 nm sections were cut using a Leica UC7 Ultramicrotome. Sections were stained with 2% UA for 12 min, followed by Reynolds’ Lead Citrate for 6 min. Images were taken on a FEI Tecnai Spirit 120kV Transmission Electron Microscope operated at an accelerating voltage of 80kV and images were acquired with a DVC1500M side-mounted camera controlled by AMT software. For each cell imaged, the cell wall thickness was measured at three points using ImageJ and the average cell wall thickness was determined. Statistical significance was determined by performing a two-way ANOVA with Tukey’s multiple comparisons in R (version 4.1.3).

### 2.13 Virulence assays and tissue culture

To assess virulence in mice, inocula were prepared by growing WT and *opi3Δ* cells in YPD overnight at 30°C, washing three times in sterile PBS (Gibco, Waltham, MA, United States), and resuspending at 4.0×10^6^ cells/ml in PBS. Three female BALB/c mice aged 4–6 weeks old (Charles River Laboratories, ON, Canada) were anesthetized intraperitoneally with 80 mg/kg ketamine and 5.5 mg/kg xylazine and inoculated with each strain by intranasal instillation with 50 μl of cell suspension (inoculum of 2×10^5^ cells per mouse). Infected mice were monitored daily post-inoculation and upon displaying signs of morbidity, the mice were euthanized by carbon dioxide anoxia. For the determination of fungal burdens in organs at endpoint, cardiac blood was retrieved and organs were excised, weighed, and homogenized in two volumes of PBS using a MixerMill MM400 (Retsch, Haan, Germany). Serial dilutions of the homogenates were plated on YPD agar plates containing 50 μg/ml chloramphenicol, and colony forming units (CFUs) were counted after incubation for 48 h at 30°C. Significance in survival assays was determined using log-rank tests and significance in fungal burden was determined using Mann-Whitney U tests in R (version 4.1.3).

Phagocytosis assays were performed using the murine macrophage-like cell line J774.A1 (ATCC, Manassas, Virginia). J774.A1 cells were grown in Dulbecco’s Modified Eagle Medium (DMEM, Gibco, Waltham, MA, United States) supplemented with 10% Fetal Bovine Serum, 100 units/ml penicillin, 100 μg/ml streptomycin, and 2 mM L-glutamine at 37°C with 5% CO_2_. J774.A1 cells used for the experiments were kept between passages 5 and 25. Macrophages were seeded at a density of 200,000 cells/ml 24 h prior to infection in 24 well clear bottom plates. On the day of infection, overnight cultures of *C. neoformans* grown in YPD media at 30°C were washed three times with PBS and opsonized with 10 μg/ml of 18b7 monoclonal antibody in serum-free DMEM for 1h at room temperature. Simultaneously, macrophages were activated with 150 ng/ml of phorbol 12-myristate 13-acetate in serum-free DMEM for 1 h prior to infection. Macrophages were then incubated with the opsonized cryptococcus for 2h at a multiplicity of infection 1:10 (macrophage:yeast) at 37°C with 5% CO_2_. Immediately after incubation, each well was washed 3 times with warmed PBS to remove extracellular *C. neoformans.* Macrophages were lysed with 200ul of distilled water, serial diluted, and plated on YPD plates to obtain a count of internalized *C. neoformans.* This was done at 2 h (T2) and 20 h (T20) post infection and the intercellular proliferation rate is defined and the ratio of cell numbers between T20 and T2. Three independent experiments were conducted, and significance was determined using a one-way ANOVA with Tukey’s multiple comparisons in R (version 4.1.3).

### 2.14 Statistics

Data were visualized and statistically analyzed using R. Statistical tests were performed by two-way analysis of variance (ANOVA) followed by a Bonferroni correction, or by an independent two sample t-test as indicated in the figure legends. P-values of ≤ 0.05 were considered significant.

## 3 Results

### 3.1 Loss of Opi3 results in choline auxotrophy

**Figure 1:**
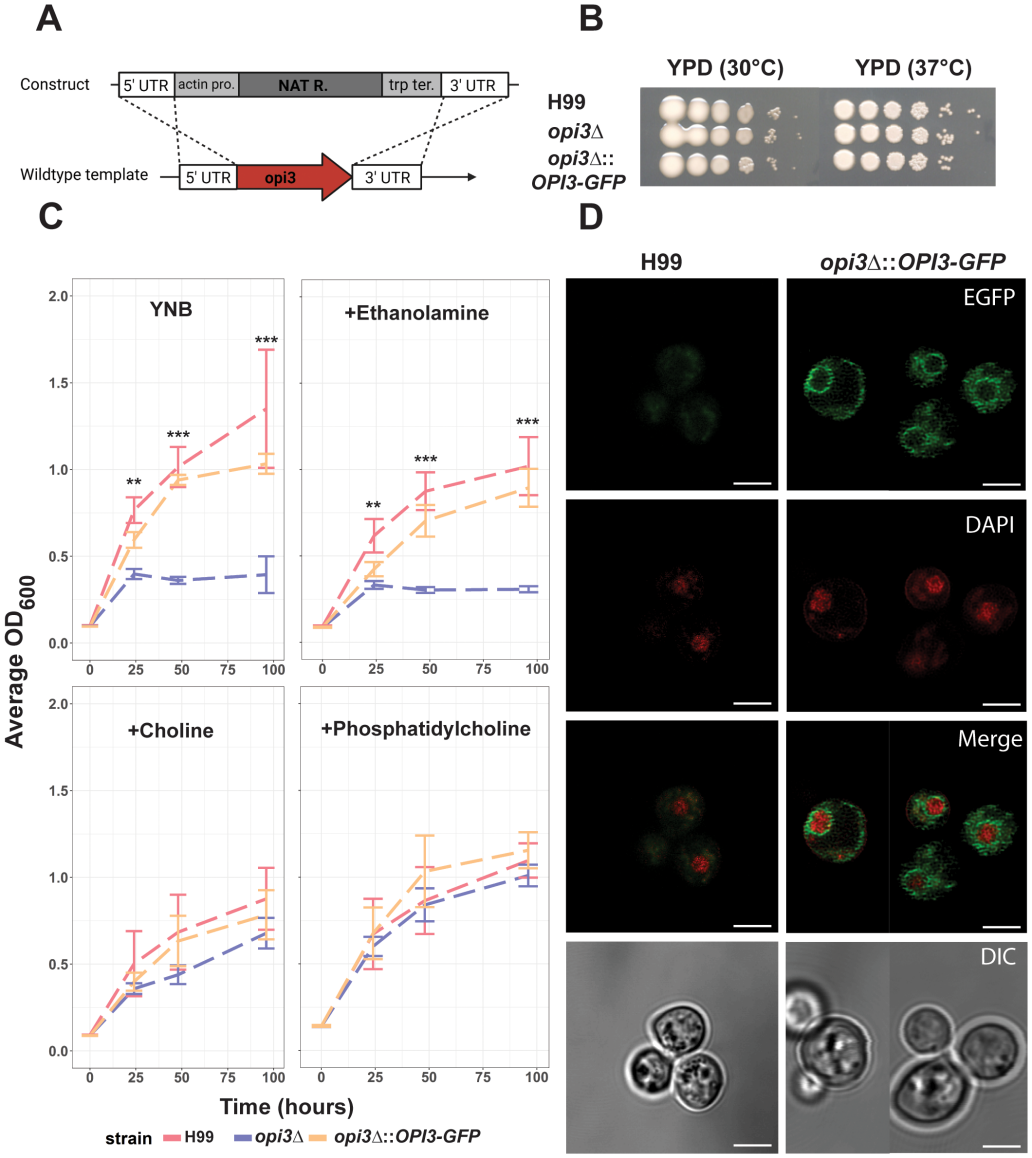
Opi3 is localized to the ER and loss causes choline auxotrophy. **(A)** The *opi3Δ* deletion mutant was generated through homologous recombination of a construct containing a nourseothricin resistance marker (NAT^R^) expressed from the actin gene promoter (pro.) and using the terminator (ter.) from the anthranilate synthase (*trp1*) gene. **(B)** Spot assays on YPD media demonstrating growth after 3 d for the WT (H99), *opi3Δ*, or the *opi3Δ*::*OPI3-GFP* strains at 30°C or 37°C. **(C)** Growth assays for the H99, *opi3Δ*, and *opi3Δ*::*OPI3-GFP* strains in liquid YNB + 2% glucose medium supplemented with either 500 μM ethanolamine, 10 μM choline chloride, or 300 μM phosphatidylcholine (PC). The error bars represent the SD of three biological replicates. Significant differences between strains were determined using a 1-way ANOVA at each timepoint (**p<0.01, ***p<0.001). The cultures were incubated at 30°C for 100 h and OD_600_ values were measured via a plate reader. **(D)** Opi3 was C-terminally tagged with GFP (green) to examine intracellular localization in parallel with DAPI staining (red) to highlight localization of Opi3-GFP in the perinuclear endoplasmic reticulum. Scale bar = 5 µm. All microscopy images are representative of minimum of 20 cells.

To investigate the role of phosphatidylcholine (PC) biosynthesis and lipid homeostasis in cryptococcal virulence, we first constructed an *opi3Δ* mutant (**Figure 1A**) and examined its phenotypes in culture (**Figures 1B, C**). We found that loss of Opi3 did not result in a growth defect in yeast-peptone dextrose (YPD) media at 30°C or 37°C (**Figure 1B**). However, the mutant exhibited a growth defect in minimal media (YNB) and reached stationary phase at a lower cell density than WT, a phenotype consistent with observations of an o*pi3Δ* mutant in *S. cerevisiae* (**Figure 1C**) (McGraw and Henry, 1989). Growth was also tested in ethanolamine, PC, or choline to confirm the role of Opi3 in PC biosynthesis in *C. neoformans*. In this case, supplementation with 10 μM choline or 300 μM PC was sufficient to rescue the growth of the *opi3Δ* mutant, while 500 μM of ethanolamine did not rescue (**Figure 1C**). Eukaryotic phospholipid N-methyltransferases are known to localize the endoplasmic reticulum (ER) and Opi3 in *S. cerevisiae* localizes with an active site orientation consistent with its proposed role in *trans* catalysis (Tavassoli et al., 2013). To localize Opi3 in *C. neoformans*, we tagged the protein with GFP at the C-terminus and determined that Opi3-GFP was found in the perinuclear ER region and at the plasma membrane (**Figure 1D**). The conclusion that the protein localized to the perinuclear ER was supported by co-staining of nuclear DNA with DAPI to detect the ring-like signal directly surrounding the stained nucleus. The Opi3-GFP fusion protein was functional as demonstrated by complementation of the mutant growth defect (**Figure 1C**), and expression of the intact protein (60.8 kDa) was confirmed by immunoblotting using an anti-GFP antibody (**Supplementary Figure S1**). Together, these results support the role of Opi3 in PC synthesis and the location of the protein in the ER and plasma membrane in *C. neoformans*.

### 3.2 Growth of the *opi3Δ* mutant is rescued by sorbitol and polyethylene glycol

**Figure 2:**
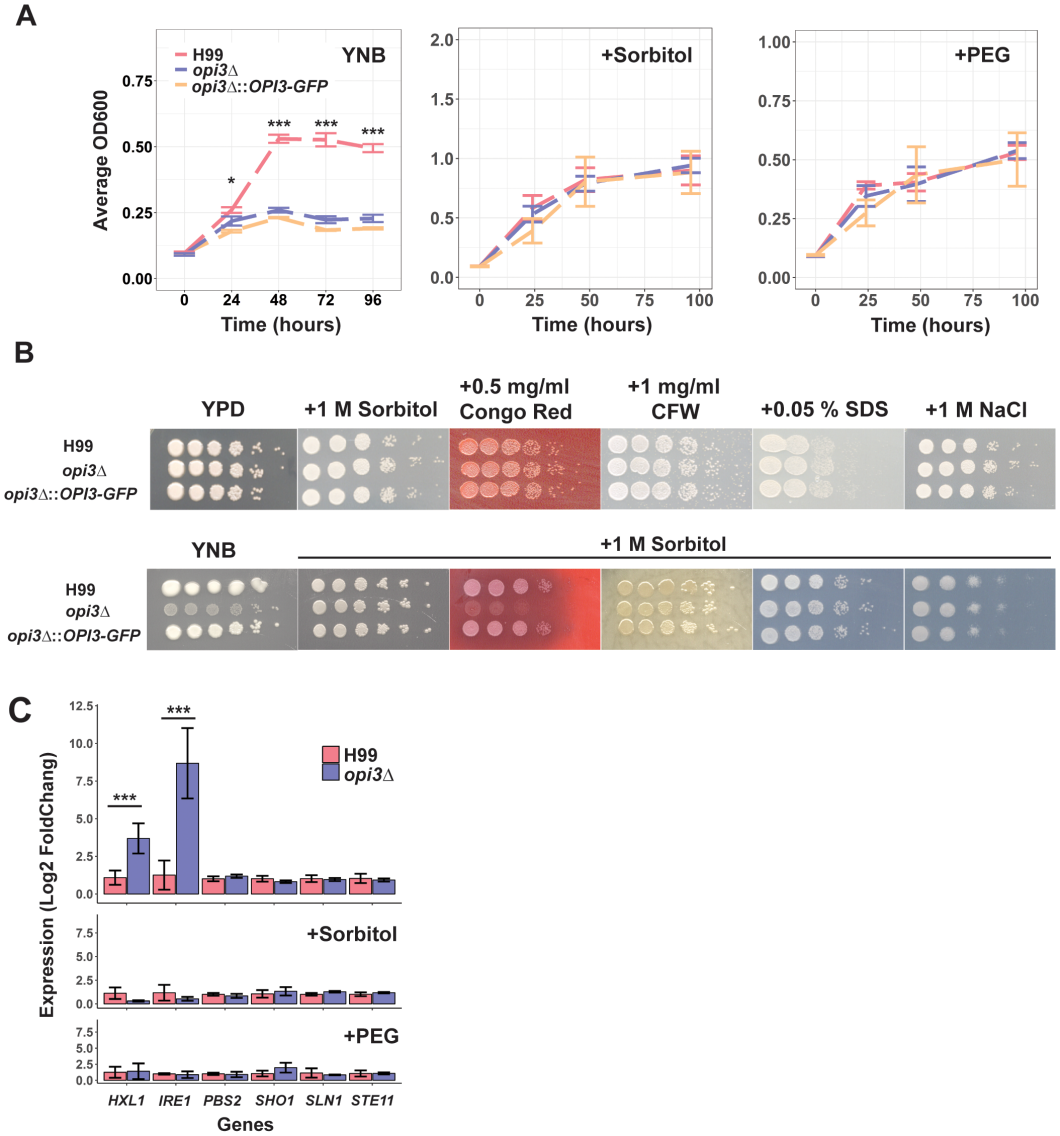
Sorbitol, polyethylene glycol and congo red influence the growth of the *opi3Δ* mutant. **(A)** Examination of growth in YNB with and without 1 M sorbitol or 10% polyethylene glycol (PEG). Strains were growth in YPD overnight before inoculating 1x10^5^ cells/ml in 96 well plates. Plates were grown at 30°C and 200rpm with multiple OD_600_ readings taken over 80 or 120 h. Error bars represent the SD between 3 biological replicates. Significance differences between strains were determined using a 1-way ANOVA at each timepoint (*p<0.05, ***p<0.001). **(B)** Examination of growth on solid rich media (YPD) with congo red and other cell wall stressors (calcofluor white (CFW), SDS, or NaCl). A parallel experiment was performed specifically on YNB medium to test the impact of 1 M sorbitol on sensitivity to congo red and the other stressors. Strains were grown overnight in YPD before normalizing cell density among strains and spotting 5 μl in 10-fold dilutions starting at 1 x 10^6^ cells on plates of the indicated stressors. Plates were incubated at 30°C for 2-3 days before scanning. Each image is representative of 3 biological replicates. **(C)** Quantitative real-time PCR of genes related to the unfolded protein response and the high osmolarity glycerol response pathway within H99 (WT) and *opi3Δ* after 24 h of growth in YNB, YNB + 1 M sorbitol, or YNB + 10% PEG. Statistical significance was determined using an independent two sample T-test (*p<0.05, ***p<0.001).

We next tested the hypothesis that the *opi3Δ* mutant had altered plasma membrane integrity due to changes in phospholipid composition. We first demonstrated that growth of the mutant was rescued in YNB with the addition of 1 M sorbitol or 10% polyethylene glycol (PEG) (**Figure 2A**). The mutant did not show significant susceptibility to agents that challenge membrane integrity or provoke osmotic stress (NaCl, SDS, mannitol, glycerol or sucrose) in rich YPD or minimal YNB media with or without 1 M sorbitol (**Figure 2B**; **Supplementary Figure S2**). Growth assays on agar medium with sorbitol further revealed that the *opi3Δ* mutant was hyper-susceptible to growth on congo red thus highlighting beta-glucans as a component of interest in cell wall changes, although the mutant was insensitive to other cell wall stressors such as caffeine or calcofluor white (**Figure 2B**). Other agents such as mannitol, glycerol, and sucrose showed no rescue of growth indicating a specific role for sorbitol and PEG, perhaps as chaperones to influence the unfolded protein response in the ER (**Supplementary Figure S2**). It is known that PC depletion is accompanied by lipid imbalances and an activation of the unfolded protein response (UPR) in *S. cerevisiae* (Vevea et al., 2015). Therefore, we examined the transcript levels for the genes encoding the major kinase *IRE1* and transcription factor *HXL1* of the UPR pathway and found that both genes were significantly upregulated in the *opi3Δ* mutant compared to the WT strain (**Figure 2C**). Moreover, this transcriptional effect was alleviated in the presence of sorbitol and PEG suggesting that these osmotic protectants acted as chaperones to influence protein unfolding in the *opi3Δ* mutant. Because sorbitol and PEG are also osmotic stressors, we investigated the transcript levels for genes of the high osmolarity glycerol (HOG) pathway including *PBS2*, *SHO1*, *SLN1*, and *STE11* (**Figure 2C**). Differences were not observed between the *opi3Δ* mutant and the WT thus suggesting an impact of sorbitol and PEG on the UPR pathway rather than the HOG pathway in the *opi3Δ* mutant (**Figure 2C**).

### 3.3 Loss of Opi3 results in lipid droplet accumulation

**Figure 3:**
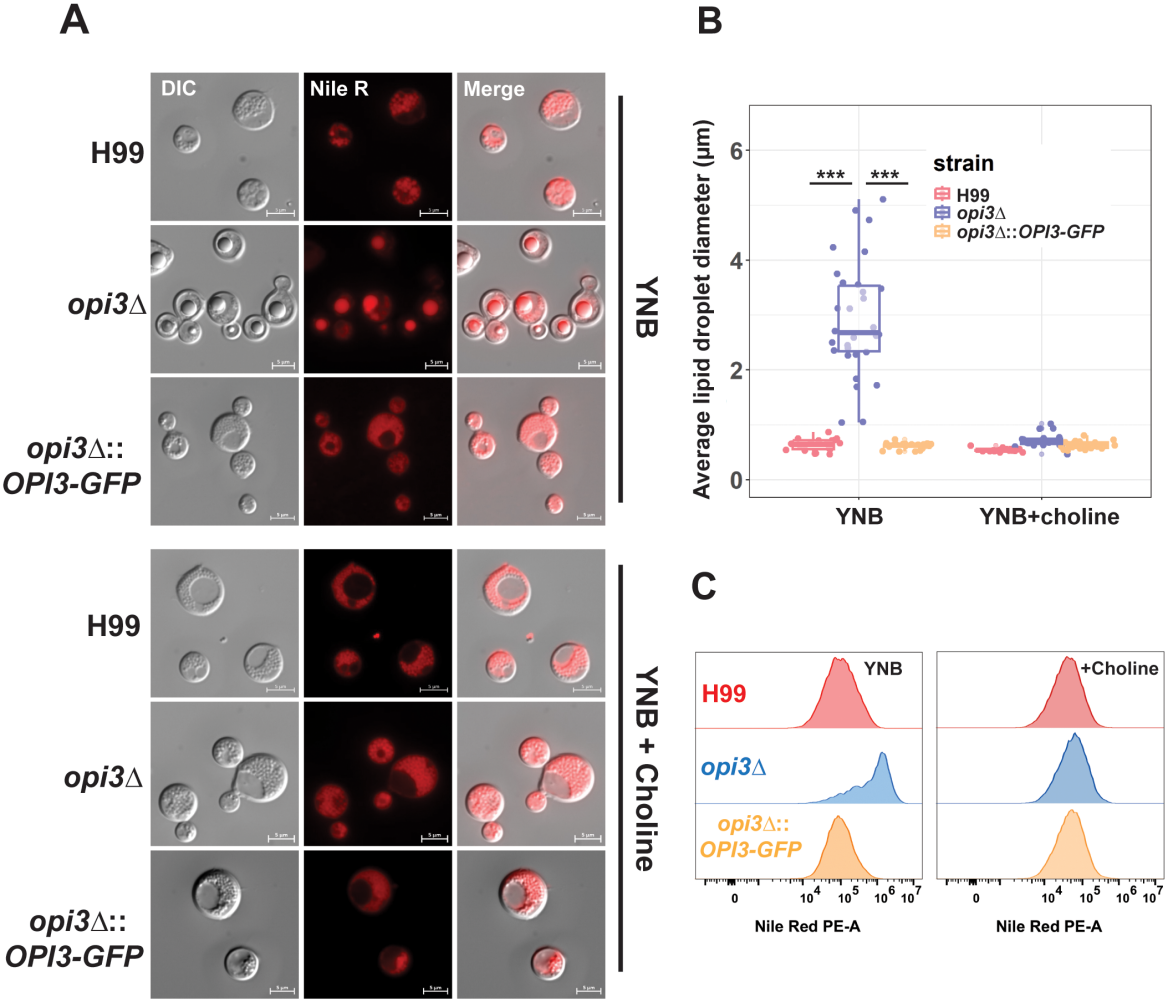
The *opi3Δ* mutant accumulates higher levels of lipid droplets compared to WT. **(A)** Representative images of cells of the H99 (WT), *opi3Δ*, and *opi3Δ*::*OPI3-GFP* strains stained with Nile Red after 48 h of growth in YNB + 2% glucose with or without 10 μM choline chloride. Scale bar = 5 μm. **(B)** Assessment of lipid droplet size in the *opi3Δ* mutant versus H99 and the complemented strain in YNB media with or without 10 μM choline chloride. The lipid droplet sizes of at least 30 cells are shown for each stain. Boxplots illustrate the 25^th^-75^th^ interquartile range while whiskers represent the bottom 25^th^ and top 75^th^ percentile. **(C)** Total triglyceride content was quantified during grown in YNB, YNB+choline, and YNB+sorbitol using flow cytometry. The distribution of 30,000 single cells/sample is shown against the PE-A channel (574 nm emission) after staining with Nile Red. Statistical significance was determined using a 2-way ANOVA with Tukey’s multiple comparisons tests (***p<0.001).

Given the role of Opi3 in PC biosynthesis, we hypothesized that *opi3Δ* would exhibit changes with lipid homeostasis, as observed previously with the mutant lacking Opi3 in *S. cerevisiae* (Wang et al., 2014; Vevea et al., 2015). We initially stained cells for triglycerides with the lipophilic stain Nile Red and found greater accumulation of large lipid droplets (LDs) in the *opi3Δ* mutant compared to WT after growth in YNB (**Figures 3A, B**). Moreover, the lipid droplets in *opi3Δ* displayed a greater intensity of Nile Red staining indicating a higher TAG content compared to WT, as revealed by flow cytometry (**Figure 3C**). The addition of choline resulted in the disappearance of the LD phenotype for the *opi3Δ* mutants, a result consistent with the observed choline auxotrophy and the importance of the Kennedy pathway as the alternate route for PC biosynthesis.

### 3.4 Phospholipid composition is altered in the *opi3Δ* mutant

**Figure 4:**
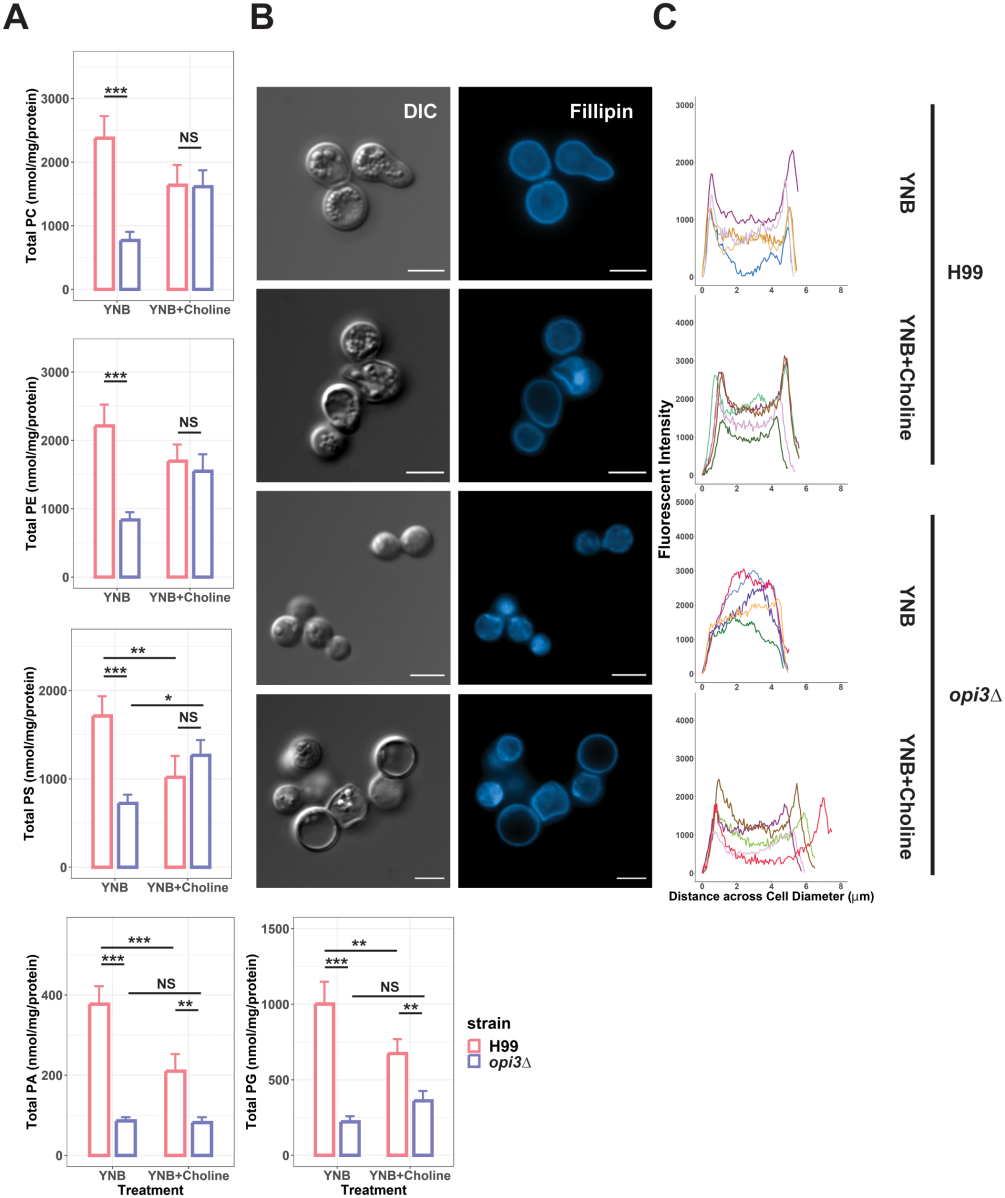
An *opi3Δ* mutant exhibits an altered membrane composition. **(A)** Graphs represent the phospholipid profiles of WT (H99) and the *opi3Δ* mutant determined by LC-MS where error bars indicate the SD of 3 biological replicates. Total phospholipid concentrations were normalized to total protein concentration. PE, phosphatidylethanolamine; PC, phosphatidylcholine; PS, phosphatidylserine; PG, phosphatidylglycerol; and PA, phosphatidic Acid. Statistical significance was determined using a 2-way ANOVA with Tukey’s multiple comparisons tests (*p<0.05, **p<0.01, ***p<0.001, NS, not significant). **(B)** Cells of WT (H99) and the *opi3Δ* mutant were stained with filipin to investigate the distribution of sterols in the membrane. Images shown are representative of at least 30 images. Scale bar = 5 um. **(C)** Intensity profiles depicting the sterol distribution across the diameter of 5 representative cells for each treatment were analysed using ImageJ.

Inhibition of phosphatidylcholine synthesis would be expected to alter phospholipid levels (Vevea et al., 2015). We therefore examined the impact of the *opi3Δ* defect in *C. neoformans* in greater detail by measuring the major phospholipid classes to further establish the contribution of *opi3Δ* to lipid homeostasis *in vitro*. The WT and *opi3Δ* strains were grown in YNB with and without choline for 24 h before isolating lipid fractions. We found that all measured phospholipid classes (PC, PE, PS, PA, and PG) had reduced levels in the *opi3Δ* mutant (**Figure 4A**) thus indicating that the deficiency in PC biosynthesis broadly affected phospholipid biosynthesis. Moreover, supplementing the same samples with choline rescued PC, PE, and PS levels in the *opi3Δ* mutant but PA and PG remained significantly depleted thus potentially highlighting the greater importance for PC, PE, and PS in growth. Interestingly, the addition of choline to the WT strain resulted in reduced concentrations of PS, PA, and PG that were still significantly higher than in the *opi3Δ* mutant. We also investigated the PC/PE ratio as a marker for lipid dysregulation but found no significant changes between the *opi3Δ* and WT strains (Janssen et al., 2000; Boumann et al., 2006) (**Supplementary Figure S3**). As another measure of membrane composition, we examined the distribution of ergosterol in the membrane for the WT and mutant strains using the ergosterol-binding dye Fillipin. With this assay, staining was clearly localized to the plasma membrane in WT with and without the addition of choline (**Figure 4B**). However, ergosterol distribution in the *opi3Δ* membrane appeared non-uniform and punctate indicating deficiencies in ergosterol deposition in the membrane. In fact, ergosterol distribution in the *opi3Δ* mutant appeared to be more prominent in the cytoplasm compared with the distribution in the WT strain (**Figure 4C**). Expectedly, the altered ergosterol distribution in the *opi3Δ* mutant was rescued with choline, as seen with other phenotypes.

### 3.5 Loss of Opi3 contributes to structural changes in the *C. neoformans* cell wall

**Figure 5:**
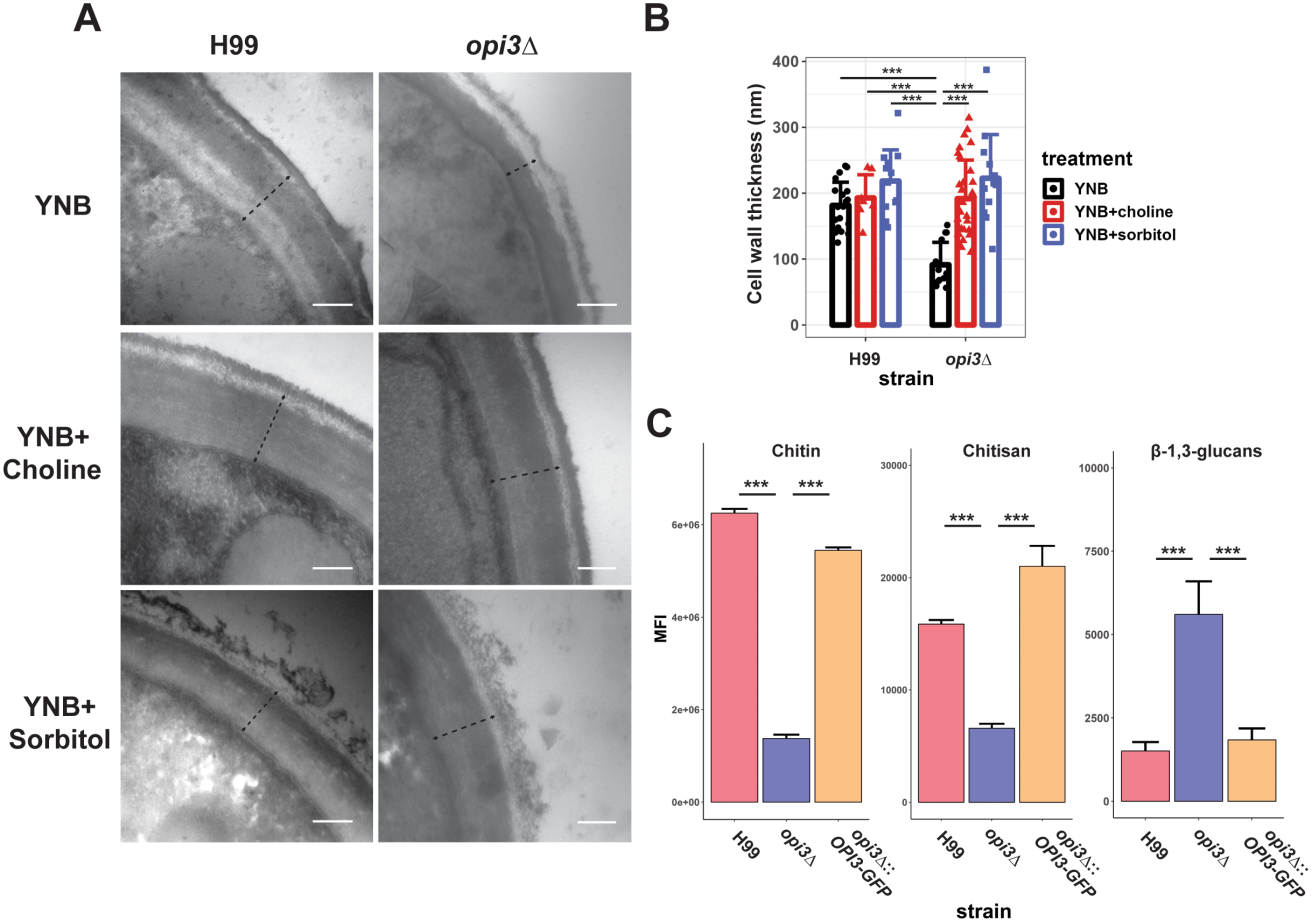
Loss of Opi3 alters cell wall composition. **(A)** Cell wall thickness from representative TEM images (98,000x magnification) with a dotted line indicating the measured size. Scale bar = 200 nm. Cell wall thickness was measured for the H99 (WT) and *opi3Δ* strains after 24 h of growth in YNB with or without 10 μM choline or 1 M sorbitol at 30°C degrees. **(B)** The cell wall thickness for each sample is shown as the average of at least 20 cells. **(C)** Relative abundance of chitin, chitosan and β-1,3-glucans in the cell wall of the WT, *opi3Δ*, and *opi3Δ*::*OPI3-GFP* strains as detected using calcofluor white, eosin Y or aniline blue stains, respectively. The mean fluorescent intensity of 30,000 cells was captured on a Attune Nxt Flow Cytometer over three independent experiments. Error bars represent SD. Statistical significance was determined using a 2-way ANOVA with Tukey’s multiple comparisons tests (***p<0.001).

We next examined the impact of the *opi3Δ* mutation on cell wall structure because of the observed sensitivity to congo red and the potential that altered membrane composition could affect cell wall integrity. First, cell wall thickness was compared between the *opi3Δ* and WT strains by transmission electron microscopy, and we found the mutant wall to be significantly thinner (**Figure 5A**). As expected, the addition of choline or sorbitol led to restoration of cell wall thickness of the *opi3Δ* mutant to the WT level (**Figures 5A, B**). No difference in cell size was observed between the *opi3Δ* mutant and the WT strain H99. Moreover, the WT strain grown in YNB medium exhibited a clear two-layered cell wall while only the outer layer was evident in the *opi3Δ* mutant. Knowing that the inner cell wall is comprised of chitin and chitosan among other components (Reese et al., 2007), we examined surface exposed chitin and chitosan content by staining with CFW and Eosin Y, respectively. Through flow cytometry, both components were reduced in the mutant compared to the WT and complemented strains thus supporting the conclusion of an altered cell wall structure in the *opi3Δ* mutant (**Figure 5C**). Furthermore, surface exposed β-1,3-glucans were measured using aniline blue and found to be at significantly higher levels in the mutant, consistent with the altered sensitivity to congo red.

### 3.6 Loss of Opi3 results in reduced cell attachment of capsule polysaccharide

**Figure 6:**
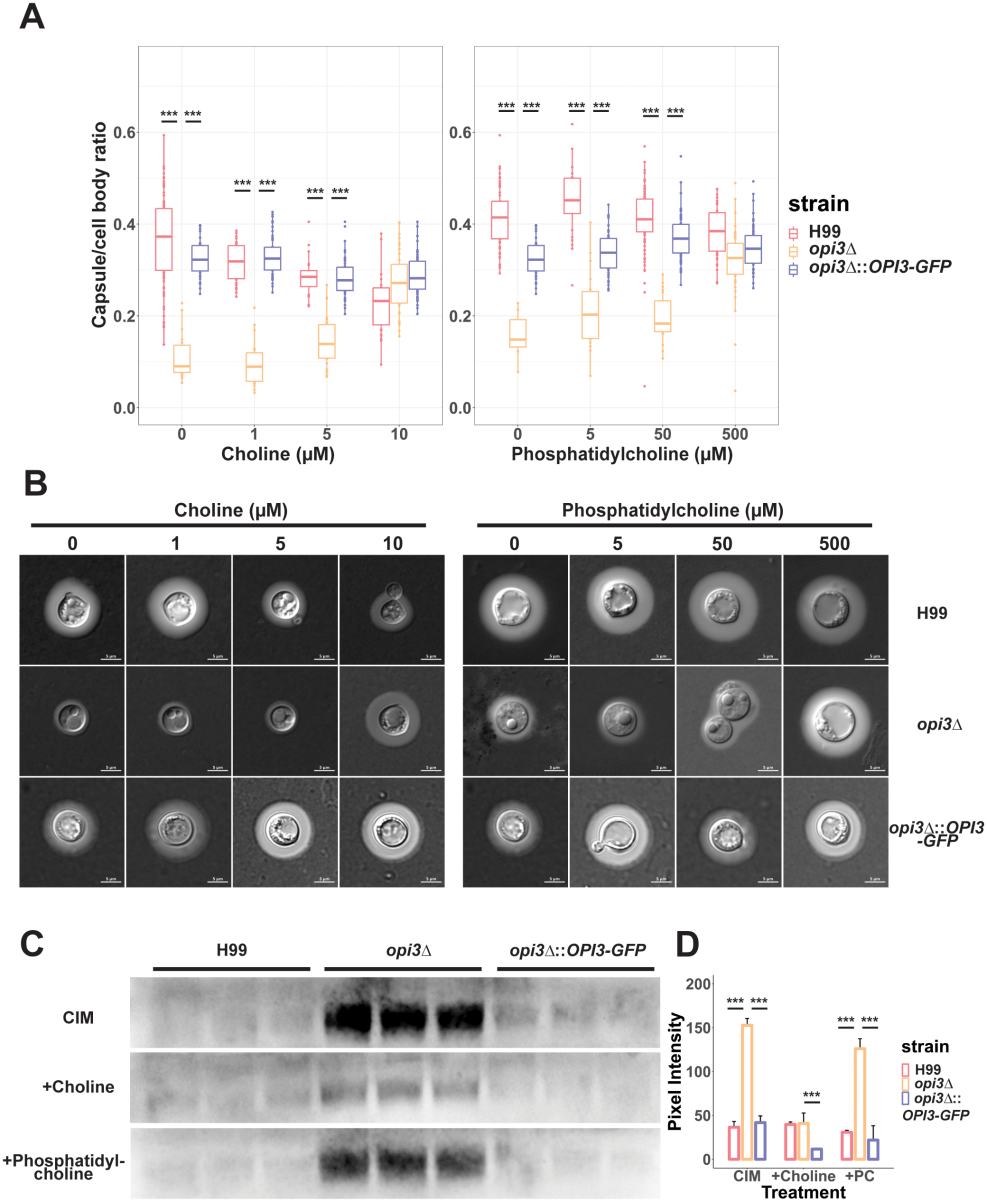
The *opi3Δ* mutant exhibits reduced capsule size and increased polysaccharide shedding. **(A)** Cells were grown in defined low iron media with 10 μM choline chloride or 300uM phosphatidylcholine for 48 h at 30°C to induce capsule formation and capsule size was measured after staining with India Ink. Each boxplot represents the average cell diameter or capsule thickness of at least 100 cells. **(B)** Representative images for WT, *opi3Δ*, and *opi3Δ*::*OPI3-GFP* strains stained with a 1:1 volume of India Ink before being imaged at 100X (DIC). Scale bar = 5 μm. **(C)** Immunoblotting with anti-capsule antibody to assess shed capsule polysaccharide for the *opi3Δ* mutant compared to the WT strain. Cultures were standardized to an OD_600_ = 2.5 to account for differences in cell density. Each lane represents a biological replicate (n=3). **(D)** Shed capsule polysaccharide was quantified through the peak area of the pixel intensity for each band and normalized against the background noise. The mean and SD are shown for each group. Statistical significance was determined using a 2-way ANOVA with Tukey’s multiple comparisons tests (***p<0.001).

Changes in cell wall composition may have an influence on elaboration of the polysaccharide capsule including attachment (Reese and Doering, 2003). When capsule elaboration was tested for the *opi3Δ* mutant in capsule inducing media, we found that capsule formation was impaired but not completely inhibited (**Figures 6A, B**). This phenotype was independent of cell size because no differences were found between the *opi3Δ*, WT or complemented strains. Consistent with the choline auxotrophy of the *opi3Δ* mutant, capsule size was also rescued in a dose-dependent manner with increasing concentrations of choline from 0 to 10 μM (**Figure 6A**). Moreover, supplementing phosphatidylcholine also rescued capsule formation at concentrations of 500 μM (**Figure 6A**). Additional capsule inducing conditions were tested and we found that the *opi3Δ* mutant exhibited a similar capsule deficiency in 125 μM mannitol but not in 10% FBS, likely due to choline available in the serum (**Supplementary Figure S4**). Since capsule elaboration was impaired for the *opi3Δ* mutant, shed capsule polysaccharide was quantified to examine whether inhibition of capsule synthesis or reduced attachment to the cell wall accounted for the reduced size. Compared to the WT and complemented strain, the *opi3Δ* mutant showed a higher level of shed capsule in capsule inducing media (**Figures 6C, D**). Moreover, adding choline significantly reduced capsule shedding in agreement with its rescue of capsule formation. The increased capsule shedding observed for the mutant is consistent with a change in the cell wall to impair polysaccharide attachment. Interestingly, supplementing the *opi3Δ* mutant with 500 μM PC did not reduce shed capsule levels despite rescuing capsule formation. This result suggests that choline and PC may influence capsule formation by different mechanisms perhaps due to differences in uptake.

### 3.7 Opi3 contributes to melanin formation in the cell wall

**Figure 7:**
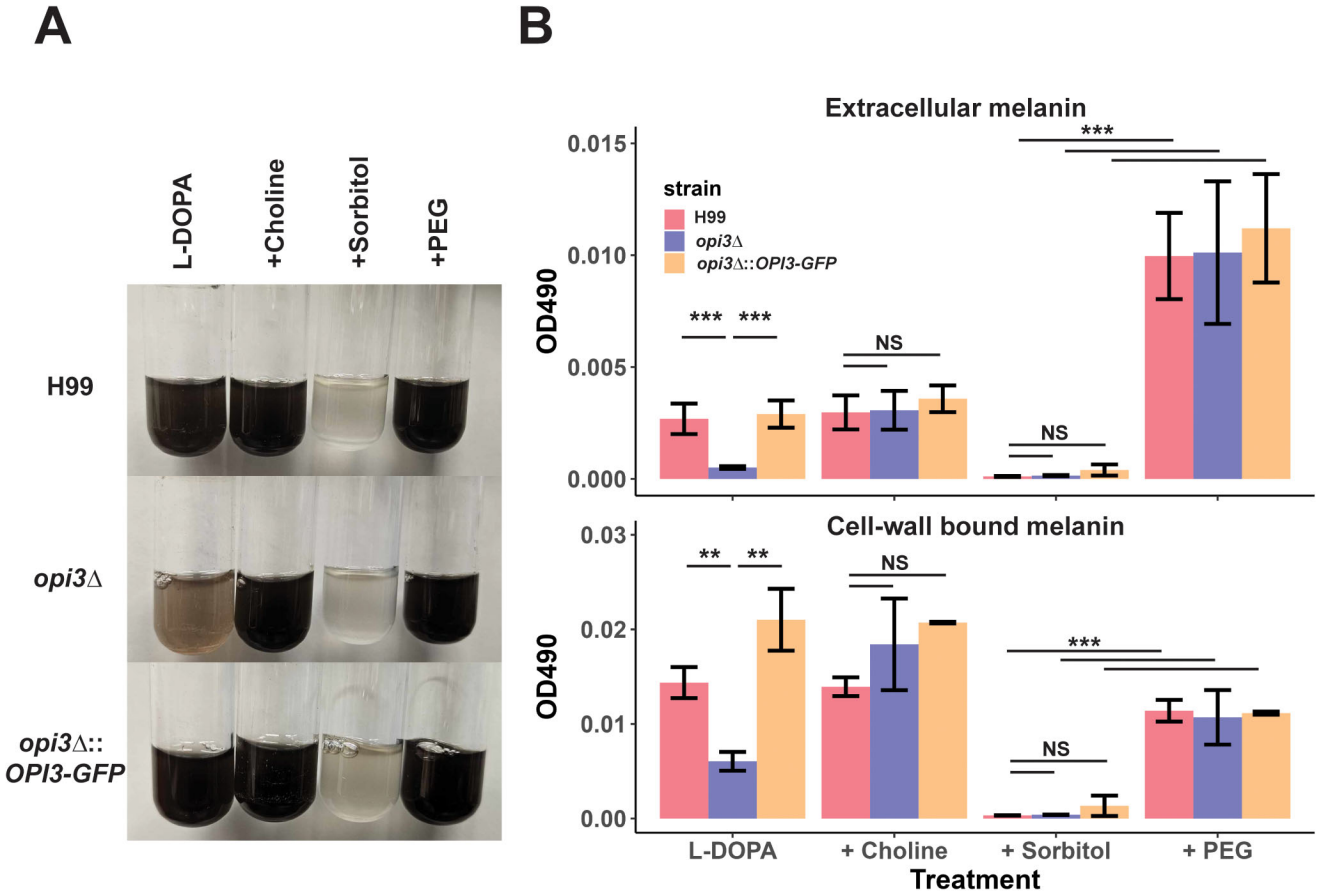
Melanin formation is impaired in the *opi3Δ* mutant. **(A)** Images of differences in melanin pigment accumulation in liquid cultures for the WT (H99), *opi3Δ*, and *opi3Δ*::*OPI3-GFP* strains. Cells were grown for 72 h in the dark in L-DOPA with 10 μM choline, 10% PEG, or 1M sorbitol at 30°C. Images were taken after 72 h of growth. **(B)** Melanin was quantified by measuring the OD_490_ from the supernatant (extracellular melanin) or the digested cell pellet (1M NAOH + 10% DMSO) (cell-wall bound melanin) and normalized to cell density. Error bars represent SD between 3 biological replicates. Statistical significance was determined using a 2-way ANOVA with Tukey’s multiple comparisons tests (**p<0.01, ***p<0.001, NS, not significant).

The differences in cell wall structure and composition for the mutant also prompted an examination of the formation of melanin, another virulence factor of *C. neoformans*. In general, the addition of 1 M sorbitol significantly reduced melanin formation while 10% PEG significantly increased extracellular melanin in the WT, *opi3Δ*, and complement strains (**Figure 7A**). Unique to the *opi3Δ* mutant, we found that extracellular and cell-wall bound melanin were significantly reduced (**Figure 7B**). Addition of choline rescued the melanin deficiently for the mutant at 30°C. It is interesting to note that while sorbitol inhibited melanin for all strains, PEG was also able to rescue melanin in the *opi3Δ* mutant suggesting that the osmotic stress plays a role in melanin formation.

### 3.8 Loss of Opi3 does not influence virulence in mice or proliferation in macrophages

**Figure 8:**
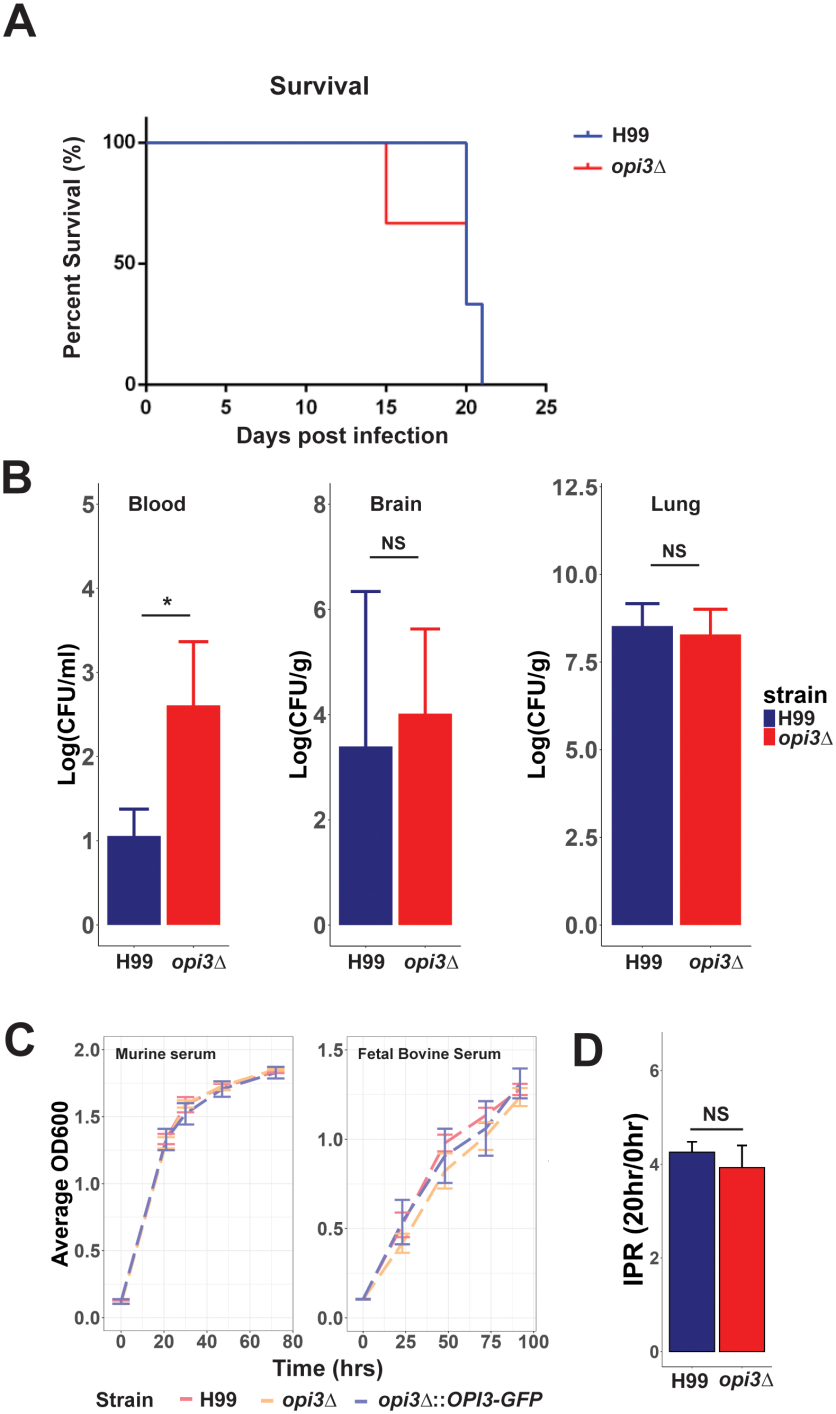
Loss Opi3 does not impact virulence in mice or intracellular proliferation in a macrophage-like cell line. **(A)** Survival of three BALB/c mice inoculated intranasally with the H99 (WT) strain or the *opi3Δ* mutant. **(B)** Fungal burdens in the blood, brain, and lungs were measured by determining colony forming units (CFU) in homogenized tissues at the humane endpoint. **(C)** Growth of the WT, *opi3Δ*, and *opi3Δ*::*OPI3-GFP* strains in (i) 5% serum extracted from BALB/c mice or in (ii) 5% heat-inactivated fetal bovine serum. The assay was performed in 96 well plates inoculated at 5x10^5^cells/ml and measured every 24 h up to 100 h. **(D)** The intracellular proliferation rate (IPR) of *opi3Δ* within macrophages exhibited no significant changes compared to the wildtype. The IPR represents a ratio of growth between 0 h and 20 h post infection. The immortalized murine macrophage-like cell line J774A.1 was used for the phagocytosis assays. Error bars represent SD between 3 biological replicates and statistical significance was determined using an independent two sample T-test (*p<0.05, NS, not significant).

The influence of the *opi3Δ* mutation on capsule and melanin formation suggested a potential decrease in virulence, and this idea was tested by intranasal inoculation of mice (**Figure 8A**). Unexpectedly, there was no difference in mouse survival between inoculation with the WT and mutant strains. However, fungal burden of *opi3Δ* in the blood was found to be increased compared to the WT while no differences were observed in the lungs or brain (**Figure 8B**). These results imply that sufficient levels of choline may be present *in vivo* to allow the mutant to proliferate and cause disease. In support of this hypothesis, the *opi3Δ* mutant was found to grow as well as the WT and complement strains in YNB with 5% murine serum or 5% heat inactivated fetal bovine serum (**Figure 8C**). These results are also consistent with an analysis of intracellular proliferation in a macrophage-like cell line that revealed no significant difference between the *opi3Δ* and WT strains in their ability to grow intracellularly (**Figure 8D**).

## 4 Discussion

In this study, we examined the role of phosphatidylcholine biosynthesis in *C. neoformans* by characterizing mutants lacking Opi3, a phospholipid N-methyltransferase (PLMT). We showed that these mutants were choline auxotrophs and had altered cell wall composition, increased shedding of capsule polysaccharide, reduced cell wall melanin, and reduced phospholipids when deprived of choline. Interestingly, we observed no virulence defect for the *opi3Δ* mutant in a murine survival assay suggesting that *C. neoformans* can rely exclusively on host sources of choline during all stages of infection. *C. neoformans* has previously been shown to have a functional Kennedy Pathway for phosphatidylcholine biosynthesis through studies of the *CHO1* gene encoding phosphatidylserine synthase (Konarzewska et al., 2019). A *cho1Δ* deletion mutant supplemented with ethanolamine or choline can produce PE and PC as demonstrated with ^14^C-labelling. This study revealed that the enzyme is essential for viability and mutant growth was not rescued by addition of choline or ethanolamine. Similarly, we found that an *opi3Δ* mutant fails to grow in YNB media supplemented with ethanolamine but could be rescued with the addition of choline thus providing further insights into the function of the Kennedy Pathway in *C. neoformans*.

Studies in *S. cerevisiae* provide a foundation for appreciating the contributions of Opi3, as the terminal PLMT within the *de novo* PC biosynthetic pathway that is localized to the ER (Pawlik et al., 2020). However, PC can also be synthesized through the Kennedy Pathway via ethanolamine or choline imported by the transporter Hnm1. In this pathway, ethanolamine is imported and converted to PE, which is methylated by Opi3 to form PC. Alternatively, choline is imported and directly used to synthesize PC bypassing the *de novo* PC biosynthetic pathway. LD formation in the context of PC deficiency has previously been investigated in *S. cerevisiae* upon deletion of *CHO2* encoding a PEMT responsible for the first methylation of PE to PC (Vevea et al., 2015). During chronic choline starvation, LD biogenesis and depletion of PE, PC, PS, PG and PA was observed in the *cho2Δ* mutant. Our results are consistent with these findings because the *C. neoformans opi3Δ* mutant also exhibited LD biogenesis after 24 h of choline starvation. Moreover, LD biogenesis was accompanied by a depletion of PE, PC, PS, PG, and PA in the mutant. One explanation is that depletion of phospholipids is tied to LD biogenesis. Vevea et al. (2015) described a model for LD homeostasis in *S. cerevisiae* that relies on the conversion of excess phospholipids to triglycerides (TAGs) by the diacylglycerol‐acyltransferases Dga1 and Lro1, with subsequent TAG storage in LDs. Importantly, inhibition of PC biosynthesis resulting in lipid imbalance influenced the unfolded protein response and the association of LDs with aggregates of the ER. In support of this model, we show that expression of the UPR pathway is upregulated in the *opi3Δ* mutant, as demonstrated by elevated transcripts for *HXL1* and *IRE1*. This finding is further supported by the discovery of a role for PMME in ER stress and *IRE1* upregulation in *S. cerevisiae* (Ishiwata-Kimata et al., 2021). Moreover, our finding that the *opi3Δ* mutant contained reduced PL levels and a significantly higher concentration of TAGs suggests that a similar model for lipid stress and homeostasis is conserved in *C. neoformans.* Similar processes likely occur in other fungi. For example, loss of Fat Storage-Inducing Transmembrane Protein 2 (*FIT2*) in *Candida parapsilosis* prevented LD formation and attenuated virulence in a murine infection model. Similarly, deletion of *PAH1* responsible for diglyceride synthesis in *Fusarium graminearum* impaired LD biogenesis and virulence (Nguyen et al., 2011). Given the findings on adaptation to lipid stress in *S. cerevisiae* (William James et al., 2019; Ishiwata-Kimata et al., 2021), it is possible that a similar adaptation upon loss of Opi3 in *C. neoformans* led to LD biogenesis and modified ER function during growth in culture.

The WT level of virulence for the *opi3Δ* mutant was somewhat surprising given the observed changes in capsule and melanin. The cryptococcal capsule is a major virulence factor contributing to cryptococcal meningoencephalitis by preventing phagocytosis of alveolar macrophages and recognition of many pathogen recognition receptors (Kozel and Gotschlich, 1982; Levitz, 2002; Zaragoza et al., 2009; Bojarczuk et al., 2016). Although we found that the *opi3Δ* mutant shed significantly more capsule polysaccharide than WT, it is possible that sufficient cell-associated material remained to support virulence. Additionally, shed capsular polysaccharides contribute immunomodulatory effects during host infection (Vecchiarelli et al., 2011), and this may further enable the *opi3Δ* mutant to cause disease. It is likely, however, that choline availability in host tissue rescued proliferation and virulence factor production to support the ability of *C. neoformans* to cause disease in mice.

Along similar lines, we found that the *opi3Δ* mutant did not show significant differences in secreted or cell-wall bound melanin in culture supplemented with choline, sorbitol, or PEG. However, the secreted and cell wall-bound melanin was significantly lower in the mutant without any supplementation suggesting problems with melanin deposition to the cell wall. Chrissian et al. (2020) describe a relationship between melanin synthesis and increased TAG and sterol esters which may shed light on the role of lipid homeostasis, LD formation and melanin synthesis, although data on phospholipids were not collected (Chrissian et al., 2020). More likely, the reduced melanin in *opi3Δ* mutant may be attributed to the structural deficiencies in the cell wall leading to poor deposition. Chitin and chitosan are important structural components in the cell wall of *C. neoformans* and specifically influence melanin deposition. That is, a triple knockout of genes encoding chitin deacetylases (Cda1, Cda2, and Cda3) showed reduced chitosan levels and an inability to retain melanin in the cell wall (Baker et al., 2007). The reduced chitin and chitosan observed in *opi3Δ* mutant provide further evidence that such components are responsible for the reduced melanin phenotype. Additional evidence for a structurally impaired cell wall was seen through an imbalance of β glucans. The *opi3Δ* mutant showed a significant increase in surface exposed β-1,3-glucans indicating increased capsular permeability and poor capsular anchoring to the cell wall. This theory is consistent with the increased capsular shedding observed by the mutant. Further cell wall structure and stability was investigated with the *opi3Δ* mutant due to the dependence of cell wall stability on the plasma membrane (Garcia-Rubio et al., 2020). Filipin was used to determine changes in the distribution or intensity of ergosterol in the membrane and the *opi3Δ* mutant was found to have significant differences ergosterol deposition around the cell membrane. Therefore, depletion of PC leads to a dysregulation of membrane sterol distribution. A recent study also showed that deletion of a sterol transporter Ysp2 in *C. neoformans* impairs membrane integrity through susceptibility to SDS and amphotericin B and attenuates virulence thus suggesting that sterols and phospholipids interact dynamically to maintain membrane homeostasis (Choy et al., 2023). Moreover, the Gcr1 transcription factor in *S. cerevisiae* was shown to regulate *OPI3* transcription and similarly resulted in decreased PC and increased TAGs upon downregulating *OPI3* (Chidambaram et al., 2022).

In summary, we show that PC biosynthesis is essential for maintaining membrane stability and has an impact on cell wall structure, melanin deposition, and capsular attachment. Despite its major involvement in PC biosynthesis and the influence on virulence factors *in vitro*, Opi3 is dispensable for cryptococcal disease. Therefore, this study highlights the adaptability of *C. neoformans* in the host environment and the reliance on host nutrients to support proliferation leading to disease.

## Data Availability

The original contributions presented in the study are included in the article/**Supplementary Material**. Further inquiries can be directed to the corresponding author.
