# Supplementary material for: Loss of Opi3 causes a lipid imbalance that influences the virulence traits of Cryptococcus neoformans but not cryptococcosis
Source: Front Cell Infect Microbiol. 2024 Aug 13;14:1448229. doi: 10.3389/fcimb.2024.1448229 (PMC11347413; doi:10.3389/fcimb.2024.1448229)
Supplement: Supplementary file 1 [file DataSheet1.pdf]

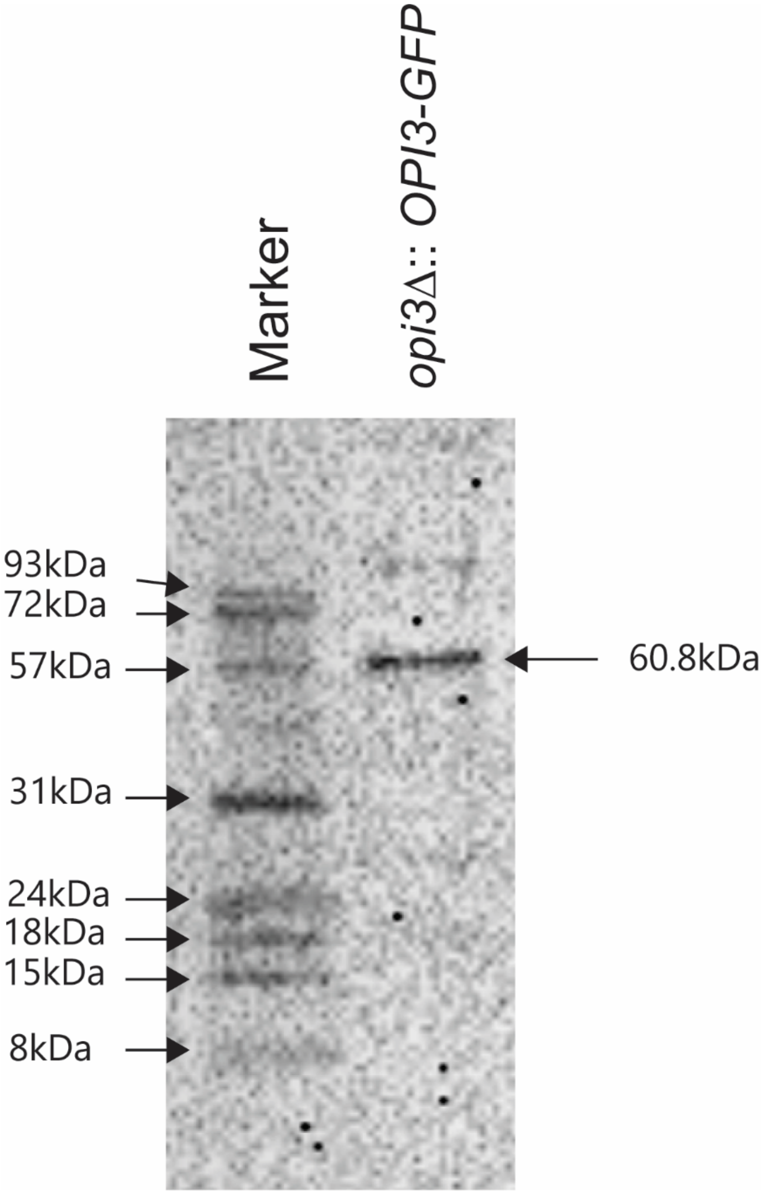

Figure S1. Immunoblot analysis of *OPI3-GFP* expression in the *opi3*Δ background. Protein lysates were separated by SDS-PAGE and probed with an anti-GFP antibody. Lane 1 shows the protein ladder, while lane 2 depicts the complement strain expressing *OPI3-GFP*. A specific band corresponding to the molecular weight of GFP (~60.8 kDa) is observed in lane 2, confirming the expression of GFP.

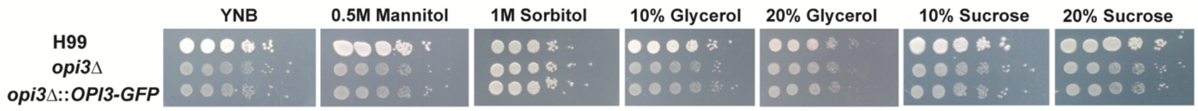

Figure S2. Examination of growth on minimal media (YNB) with sorbitol and other osmotic stressors (mannitol, glycerol, and sucrose). Strains were grown overnight in YPD before normalizing for cell density and spotting 5 µl in 10-fold dilutions starting at 1 x 10^6 cells on media with the defined stressors. Plates were incubated at 30°C for 2-3 days before scanning. Each image is representative of 3 biological replicates.

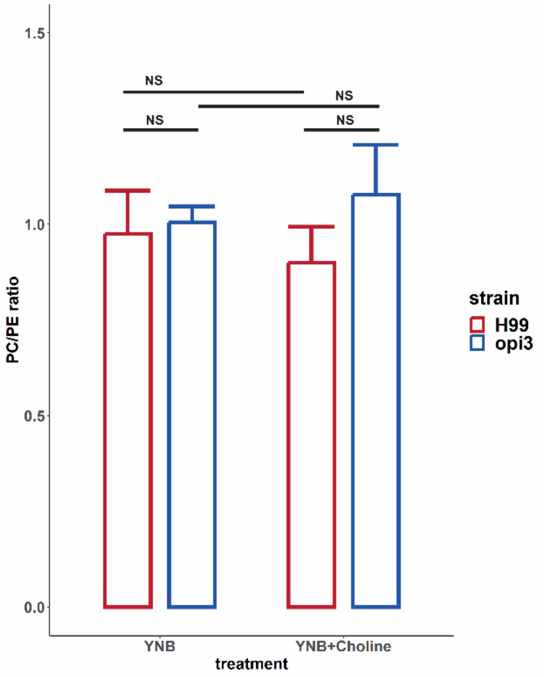

Figure S3. PE/PC ratio of H99 and *opi3*Δ in YNB or YNB + Choline. Phospholipids were measured by LC-MS where error bars represent the standard deviation of 3 biological replicates. Total phospholipid concentration was normalized to total protein concentration. PE: phosphatidylethanolamine, PC: phosphatidylcholine. Statistical significance was determined using a 2-way ANOVA with Tukey's multiple comparisons tests (NS, not significant).

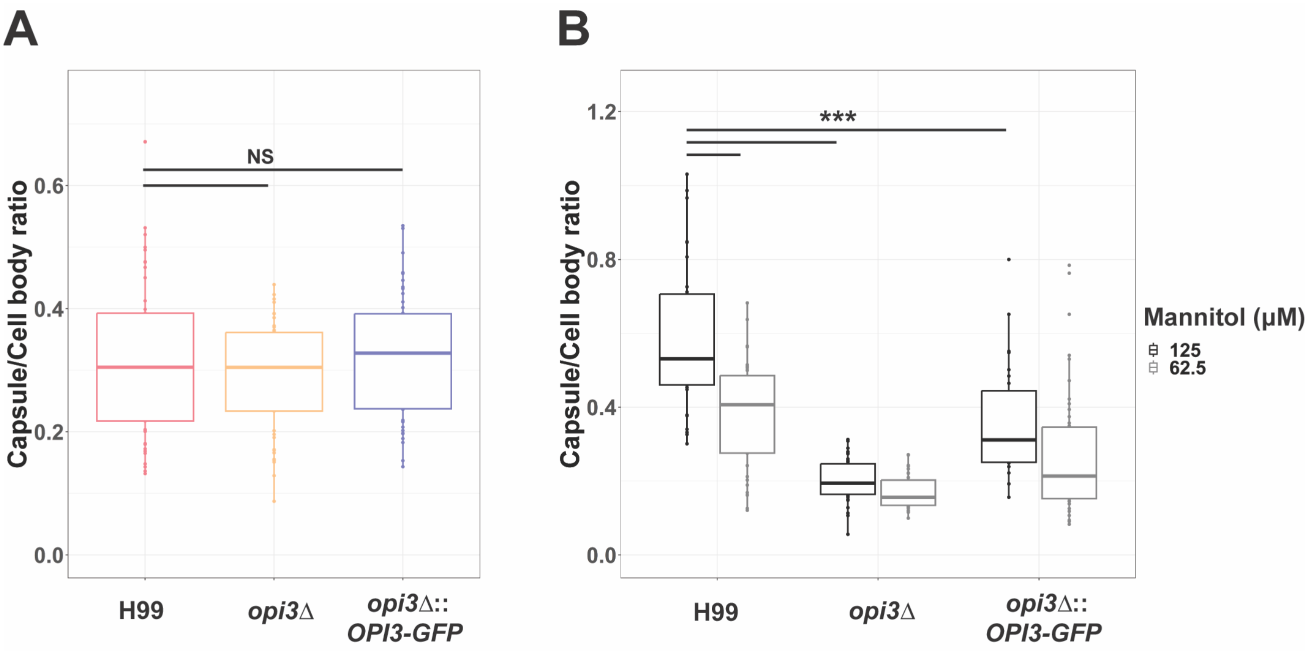

Figure S4. The reduced capsule size of *opi3*Δ is dependent on capsule inducing condition. Cells were grown in (A) 10% FBS with 5% CO_2 or (B) minimal media containing mannitol (62.5 μM and 125 μM) for 48 h at 30°C to induce capsule formation. Images were taken using a Zeiss Plan-Apochromat 100x/1.46 oil lens on a Zeiss Axioplan 2 microscope before measuring the capsule size with India Ink on ImageJ. Each boxplot represents the average cell diameter or capsule thickness of at least 50 cells. Statistical significance was determined using a 2-way ANOVA with Tukey's multiple comparisons tests (* p<0.05, ** p<0.01, *** p<0.001, NS = not significant).

Supplementary Table S1. Primers used in strain construction. The primer names and sequences used to generate each construct are listed. The templates listed in the third columns provide information on whether this part of the construct was amplified from gDNA or from a plasmid. In the final column, the primer pair for each primer is specified.

Primer Name	Sequence 5'-3'	Plasmids and templates	Primer pair
Opi3-1	gtatgttttaccagagttagtc	*opi3Δ* (Kn99)	Opi3-2
Opi3-2	ttgtcaatgccagaattcaccg	*opi3Δ* (Kn99)	Opi3-1
Opi3-GFP-1 (fragment 1)	tttcccattcaacgttctttccg	H99	Opi3-GFP-2
Opi3-GFP-2 (fragment 1)	ctcctcgcccttgctcacctcctccccactaataccactag	H99	Opi3-GFP-1
Opi3-GFP-3 (fragment 2)	tattagtggggaggagtagGTGAGCAAGGGCGAGGAG	pWH091	Opi3-GFP-4
Opi3-GFP-4 (fragment 2)	gctcgacgttgtcactgaagc	pWH091	Opi3-GFP-3
Opi3-GFP-5 (fragment 3)	ccatgggtcacgacgagatc	pWH091	Opi3-GFP-6
Opi3-GFP-6 (fragment 3)	ccagttccacaacttcaaggtggttgtaaaacgacggccagtg	pWH091	Opi3-GFP-5
Opi3-GFP-7 (fragment 4)	cactggccgtcgttttacaaccaccttgaagttgtggaactgg	H99	Opi3-GFP-8
Opi3-GFP-8 (fragment 4)	agggattgaacctgcgacc	H99	Opi3-GFP-7
Opi3-GFP-9	ccgatcccatgtatgttggctc	Fragment 1+2	Opi3-GFP-10
Opi3-GFP-10	ggaagggactggctgctattg	Fragment 1+2	Opi3-GFP-9
Opi3-GFP-11	ggaagggactggctgctattg	Fragment 3+4	Opi3-GFP-12
Opi3-GFP-12	ccgattaagccaaccgaggc	Fragment 3+4	Opi3-GFP-11

Supplementary Table S2. List of RT-qPCR primers used in the study.

Gene	5'-Forward Primer-3'	5'-Reverse Primer-3'
HXL1	cactccattcctttctgc	cgtaactccactgtgtcc
IRE1	tgcagaagatggcgttgc	acactcccgcctttatac
PBS2	ttacccctacccaccggag	aaccatgtcaacttctgcgc
SHO1	tctgcgcgagaggaatacaa	cgaacttggaaccacttgcc
SLN1	tatcaaccccgcgactcaat	atactccttgttcctcccgc
STE11	tcctgctccaccctctgata	tcttctacgacttgcacttcca
